# Supplementary material for: GRNContext: an interactive web platform for contextualized gene regulatory networks visualization across human cancers
Source: Bioinformatics. 2026 Jun 13;42(8):btag389. doi: 10.1093/bioinformatics/btag389 (PMC13474810; doi:10.1093/bioinformatics/btag389)
Supplement: Supplementary file 1 [file btag389_supplementary_data.zip › 1.docx]

**GRNContext: An Interactive Web Platform for Contextualized Gene Regulatory Networks Visualization Across Human Cancers.**

Ignacio Pezoa-Soto^1,2,3^, Sergio Hernández-Galaz^1,4^, Javier de las Rivas^5,6^, Alvaro Lladser^1,4^, Manuel Varas-Godoy^1,2,*^, Alberto J.M. Martin ^1,3,*^.

(1) Centro Basal Ciencia & Vida, Santiago, Chile. (2) Centro de Biología Celular y Biomedicina (CEBICEM), Facultad de Ciencias, Universidad San Sebastián, Santiago, Chile. (3) Escuela de Ingeniería, Facultad de Ingeniería, Universidad San Sebastián, Santiago, Chile. (4) Facultad de Medicina, Universidad San Sebastián, Santiago, Chile. (5) Cancer Research Center (CiC-IBMCC, CSIC/USAL), Consejo Superior de Investigaciones Científicas (CSIC)/University of Salamanca (USAL), Salamanca, Spain. (6) Instituto de Investigación Biomédica de Salamanca (IBSAL), Salamanca, Spain. . *** Corresponding author. E-mail:** [alberto.martin@uss.cl](mailto:alberto.martin@uss.cl); [manuel.varas@uss.cl](mailto:manuel.varas@uss.cl)

**Abstract**

While current Gene Regulatory Network (GRN) databases provide comprehensive reference maps of potential interactions between transcription factors and target genes, they do not specify which regulatory interactions are active within specific biological contexts. This limitation is particularly critical in cancer, where transcriptional programs are inherently tissue-specific. To address this gap, we developed **GRNContext**, an interactive web platform designed for the visualization, exploration, and comparative analysis of gene regulatory networks contextualized across 33 cancer types from The Cancer Genome Atlas (TCGA). Our approach uses the TFLink human reference GRN as a starting point and integrates TCGA transcriptomic profiles to infer cancer-specific regulatory activity. Regulatory relevance was assessed using complementary machine learning and statistical methods, which were unified into a consensus score to prioritize and filter the most relevant candidate regulators for each target gene. By providing both curated context-specific GRNs and a user-friendly platform, GRNContext constitutes a comprehensive and accessible resource that supports mechanistic investigations, hypothesis generation, and translational research focused on transcriptional regulation in cancer.

**Availability:** Freely available on the web at <https://apps.cienciavida.org/grncontext> with all major browsers supported.

**1. Introduction**

Transcriptional regulation in mammalian cells is a fundamentally context-dependent process, where gene expression is driven by the precise coordination of cell-type-specific transcription factor (TF) profiles [[1–4]](https://paperpile.com/c/AlC1NU/SNNdx+iYsWk+ZTlEN+OuIdN). This regulation relies on the existence of accessible, nucleosome-depleted chromatin that enables regulatory proteins to interact with DNA, a state that is itself highly specific to unique lineages and developmental stages [[5–9]](https://paperpile.com/c/AlC1NU/IQtx5+I17sU+SrPK6+sLLn9+wUte9). Furthermore, this complex machinery is physically constrained by a dynamic 3D genome architecture. The spatial organization of chromatin into compartments and topologically associating domains restricts TF–promoter interactions, ensuring that gene expression remains tightly linked to the unique identity, signaling state, and differentiation context of each cell [[2,5,7,10,11]](https://paperpile.com/c/AlC1NU/iYsWk+SrPK6+ZuF9o+LCSaG+IQtx5). Notably, Gene Regulatory Networks (GRNs) were developed to describe the set of interactions through which TFs can regulate the expression of their target genes [[12]](https://paperpile.com/c/AlC1NU/RvpPo). These networks are essential for understanding how cells maintain identity, differentiate, respond to signals, and undergo pathological changes such as cancer [[13–16]](https://paperpile.com/c/AlC1NU/SrOTI+vg5HI+G0S8u+sWAFa). Accurate reconstruction of GRNs is essential to understand molecular mechanisms that drive both normal physiology and disease progression. However, despite the growing availability and use of GRNs, most existing databases are constructed by integrating regulatory evidence from diverse experimental systems, cell types and biological conditions [[17–22]](https://paperpile.com/c/AlC1NU/S1ON8+ap2Ao+QJOxN+r9XLr+PRWZU+dZZtW). As a result, these databases provide comprehensive reference networks that try to capture all the potential TF-Target interactions in all the human genome. While these resources are valuable, they indicate which regulatory interactions are possible, but not in which context they are active. This limitation is particularly relevant in cancer, a leading cause of death worldwide, where transcriptional programs are affected in a tissue-specific manner [[23,24]](https://paperpile.com/c/AlC1NU/bYYdZ+OTshK).

To address this, few specialized resources have been developed to provide context-specific GRNs for cancer data. Notable examples include the GRAND database [[25]](https://paperpile.com/c/AlC1NU/I4I6), aracne.networks package [[26]](https://paperpile.com/c/AlC1NU/hepxx), and MOMA [[27]](https://paperpile.com/c/AlC1NU/o5XRT). These resources are very valuable, as they leverage large-scale datasets such as The Cancer Genome Atlas (TCGA) [[28]](https://paperpile.com/c/AlC1NU/aPCr) to generate context-specific regulatory hypotheses. However, their primary mode of accessibility presents a significant hurdle for a large segment of the scientific community. Specifically, these resources are predominantly distributed as computational packages or presented as complex datasets. Consequently, the effective utilization and interpretation of the powerful data within these tools requires a considerable level of bioinformatics expertise. This requirement often presents a significant barrier for experimental biologists, who are best positioned to validate these computational predictions and translate them into clinical insights. Experimentalists frequently lack the specialized programming skills or the computational infrastructure required to install, run, and properly analyze the outputs of such sophisticated packages. Therefore, there remains a need for resources that not only continue to provide the foundation of robust, context-specific networks but must also prioritize high accessibility and ease of use. The goal is to democratize access to this valuable regulatory information, ensuring it is readily available and interpretable for the broader scientific community, thereby accelerating hypothesis generation, experimental design, and the overall pace of discovery in complex diseases.

Following this line, we developed a strategy to infer tissue-specific GRNs across all 33 cancer types available in TCGA using TFLink human reference GRN as a starting point [[29]](https://paperpile.com/c/AlC1NU/zs8ay). TFLink compiles evidence from 10 different databases, and it adopts a transcription factor definition that transcends the classic vision based solely in DNA binding domains, categorizing any protein that demonstrates to participate in the regulation of gene expression as a TF. By integrating this human reference GRN with large-scale transcriptomic profiles from TCGA, we evaluated the regulatory relevance of each possible TF-Target pair using complementary statistical and machine learning approaches. This combination of metrics enabled us to create a consensus score to rank and filter the best regulators for each target gene in all 33 types of cancers in TCGA. As a result, we generated curated cancer-specific GRNs that prioritize the most informative transcription factors for each target gene.

In this article, we present GRNContext (Figure 1), an interactive web platform for the visualization, exploration, and comparative analysis of contextualized gene regulatory networks across cancer types. GRNContext enables users to query individual genes of interest, inspect the TFs that most effectively account for the target gene's expression, and evaluate regulatory patterns in different tumor contexts through an intuitive interface. Together, the curated GRNs and the GRNContext platform constitute a comprehensive and accessible resource that supports mechanistic investigations, hypothesis generation, and translational research aimed at understanding transcriptional regulation in cancer.

Figure 1. GRNContext web platform. The top panel shows the summary of the methodology used to construct the GRNs contextualized for cancer. The middle and bottom panels show the usage of the web platform.

**2. Methods**

**2.1 Transcriptomic Data**

All available bulk RNA-Seq raw transcriptomic count matrices were downloaded from TCGA using the TCGA biolinks package [[30]](https://paperpile.com/c/AlC1NU/fvHny) (9,927 samples spanning 33 different cancer types) and normalized using the Trimmed Mean of M-values (TMM) method implemented in edgeR [[31]](https://paperpile.com/c/AlC1NU/i7khA). We excluded all genes with a median normalized expression value below 10 from the analysis.

**2.2 Gene Regulatory Network**

The human reference GRN was constructed using the *Homo sapiens* small and large-scale interaction data from the TFLink database. This reference GRN encompasses 19,599 nodes, including 1,605 TFs, and features over 6.7 million edges.

**2.3 Regulatory inference**

We developed a computational pipeline to identify which TFs regulate each gene in a cancer-specific manner, similar to the one implemented by Cuesta-Atroz *et al*. [[32]](https://paperpile.com/c/AlC1NU/BfuBu). This approach combines Random Forest (RF) regression with Spearman’s correlation analysis. For each gene in each cancer type, we first identified its known regulatory TFs from the TFLink database. We then built an individualized RF Regressor model for that gene, using the expression levels of its known TFs as input features and the gene's own expression as the output to predict. The RF algorithm generates feature importance scores that quantify how much each TF contributes to predicting the target gene expression, ranking the explanatory power of each regulator. In parallel, we calculated Spearman's correlation coefficient between each TF and its target gene. This metric captures both the strength and direction of their monotonic relationship: positive correlations suggest the TF activates the gene, while negative correlations may indicate repression. A consensus regulatory score was calculated for each TF-target pair by applying Z-Score normalization and min-max scaling (0-1) to both the RF importance scores and the Spearman correlation values before combining these two complementary measures. This metric provides a unified measure of regulatory influence. The closer the TF consensus score is to 1, the more effective it is at explaining target gene expression.

To objectively select the most influential TFs for each target gene, we applied an automated selection procedure based on the elbow method, which identifies the optimal number of regulators per gene according to their consensus scores. Additionally, we generated six filtered versions of each network with varying stringency levels, retaining only the top 5, 10, 20, 30, 40, or 50 TFs per target gene based on their consensus scores.

Finally, we computed the conservation of all the regulations across the 33 TCGA tissues. The regulatory conservation metrics are available in the web platform edge table and network visualization, as well as in the Supplementary Materials.

**2.4 Web Server**

GRN Context is implemented as a client-server web application. The backend, built with FastAPI (Python), parses pre-computed contextualized GRNs stored as SIF files and exposes them through a REST API. The frontend, developed in React with Cytoscape.js for network visualization, enables interactive exploration of tissue-specific subnetworks. Both components are containerized using Docker and orchestrated via Docker Compose, ensuring cross-platform compatibility and straightforward deployment.

**3. Usage Guide and Use Cases**

To initiate an analysis, users should access the GRNContext web server and input a gene name into the "Gene focus" field. Then, check which cancers express that gene and select a TCGA cohort to work on. Following selection, the network density should be defined using predefined thresholds. While TopN thresholds (5, 10, 20, 30, 40 and 50) are recommended for hypothesis generation, the Elbow method is encouraged for identifying data driven cutoffs. The resulting network visualization displays the target gene and its most probable regulators, where activator-like interactions are represented by arrowheads and repressor-like interactions by T-shaped inhibitory heads. A higher TF consensus score indicates greater efficacy in explaining the expression of the target gene. Users may apply visual overlays to encode edge importance, consensus scores, or cross-cancer conservation metrics, thereby enhancing the interpretability of local and systemic regulatory patterns. For critical evaluation of regulatory robustness, researchers should utilize the conservation popup, accessed by selecting specific edges, to assess the frequency of TF-Target pairs across various cancer states. To inspect broader regulatory landscapes, investigators may right-click regulator nodes to pivot the query and explore upstream subnetwork contexts. Finally, it is imperative that all findings are verified using the provided tabular data below the network visualization, which details specific statistical scores. When documenting these results for publication, researchers are encouraged to consistently report the chosen cancer cohort, target gene, TF threshold, and network visualization settings.

To illustrate the capabilities of the GRNContext platform, we consider a pertinent example using genes known for their frequent overexpression across a spectrum of cancers. Specifically, we focus on two genes: PTTG1 (Pituitary Tumor-Transforming Gene 1) and RAB31 (RAB, Member RAS Oncogene Family-Like 31). PTTG1 is a well-characterized oncogene primarily responsible for regulating sister chromatid separation during the mitotic phase of the cell cycle [[33]](https://paperpile.com/c/AlC1NU/GiaJY). RAB31 is a small GTPase belonging to the RAB family. It has been implicated in the biogenesis of exosomes and linked to chemoresistance [[34,35]](https://paperpile.com/c/AlC1NU/GfZV3+mz4vD). Using GRNContext, we queried the database to identify the key transcriptional regulators of these two genes in specific cancer contexts.

**3.1 Case 1: PTTG1 in Liver Hepatocellular Cancer (TCGA-LIHC)**

The top 5 regulators displayed by GRNContext included FOXM1 and E2F1 (Figure 1, Step 3). Numerous studies have established that both FOXM1 and E2F1 are key TFs that regulate PTTG1 expression in various diseases, particularly in the context of hepatocellular carcinoma [[36–40]](https://paperpile.com/c/AlC1NU/lfwyX+uV9nF+Ip9YW+oZYfH+0ml81). The platform's ability to successfully retrieve and prioritize these well-established regulators validates its efficacy in identifying biologically relevant gene regulatory relationships. PTTG1 is expressed in 27 of the 33 TCGA cancer types, highlighting its widespread involvement in cancer. Moreover, its regulation is highly conserved, as the top transcription factors identified in LIHC are repeatedly observed across other tumors, with H2AZ1 appearing in 24 cancers, E2F1 in 12, FOXM1 and MYBL2 in 11 each, and MCM7 in 7. This consistency suggests that PTTG1 is controlled by a core pan-cancer regulatory network. To provide statistical context, we evaluated the global landscape of regulatory conservation across all 33 TCGA cancer types using the same top 5 TFs per gene threshold. Within the total universe of 835,367 edges, 571,784 of them (68.45%) are unique to a single type of cancer, indicating that the majority of regulatory interactions are highly cancer-specific. In contrast, only 1,085 edges (0.13%) are conserved in 24 or more TCGA cancer types. Consequently, the regulation of PTTG1 by H2AZ1 in 24 tumor types represents a statistically exceptional interaction, placing it within the top 0.13% most conserved regulations across all TCGA cancers. This rare degree of pan-cancer preservation underscores the robustness of this specific regulatory framework compared to the global network average, where most interactions lack such widespread conservation.

**3.2 Case 2: RAB31 in Ovarian Cancer (TCGA-OV)**

At the time of this analysis, no direct published evidence has specifically demonstrated the regulation of RAB31 in ovarian cancer. Nevertheless, recent studies indicate that RAB31 may contribute to ovarian cancer invasion via RAB31-dependent MMP secretion [[41]](https://paperpile.com/c/AlC1NU/ehTQ). The ability of GRNContext to predict regulators across different biological contexts using TCGA expression profiles is exemplified by the relationship involving RUNX1 and RAB31. Although not in ovarian cancer, experimental data confirms that RUNX1 regulates the expression of RAB31 in megakaryocytes [[42]](https://paperpile.com/c/AlC1NU/GEc4N). This cross-contextual evidence provides a strong mechanistic hypothesis generated by GRNContext, suggesting that the RUNX1-RAB31 regulatory axis may be conserved and functionally relevant in ovarian cancer, making further experimental validation necessary. RAB31 is expressed in all 33 TCGA cancer types, indicating its ubiquitous presence across human tissues. However, in contrast to PTTG1, the transcriptional regulation of RAB31 in ovarian cancer shows limited conservation across other tumor types. Only a few transcription factors are recurrently observed, with RUNX1 appearing in six cancers, and RUNX2, ETS1, and SPI1 in five, suggesting a more context-dependent regulatory network for RAB31.

**4. Future Plans**

The development of GRNContext will focus on expanding its contextual coverage by integrating additional large-scale transcriptomic resources beyond TCGA. Planned extensions include the incorporation of GTEx data to characterize gene regulatory networks in normal tissues [[43]](https://paperpile.com/c/AlC1NU/jAJPI), ARCHS4 to incorporate diverse organ-level expression profiles of human and mouse [[44]](https://paperpile.com/c/AlC1NU/KbFPk) and the development of a new protocol to generate single cell GRNs. These additions will enable more comprehensive, multiscale exploration of regulatory mechanisms, enhancing the utility of GRNContext for both basic and translational research.

**Funding**

This work was funded by Centro Basal Ciencia & Vida, FB210008 from ANID - Agencia Nacional de Investigación y Desarrollo. ANID Fondecyt projects to ALl [1251312], MVG [1230983] and AJMM [1231629]. ANID postdoctoral fellowship to SHG [3260791], and PhD fellowship to IPS [21211080]. Powered@NLHPC: this research was partially supported by the supercomputing infrastructure of the NLHPC (CCSS210001).

**Availability**

The source code employed in this work, along with the resources for the GRNContext web platform, is accessible at https://doi.org/10.5281/zenodo.18355998.

**References**

1.	[Field A, Adelman K. Evaluating Enhancer Function and Transcription. Annu Rev Biochem. 2020;89: 213–234.](http://paperpile.com/b/AlC1NU/SNNdx)

2.	[Heinz S, Romanoski CE, Benner C, Glass CK. The selection and function of cell type-specific enhancers. Nat Rev Mol Cell Biol. 2015;16: 144–154.](http://paperpile.com/b/AlC1NU/iYsWk)

3.	[Miyazaki K, Miyazaki M. The Interplay Between Chromatin Architecture and Lineage-Specific Transcription Factors and the Regulation of Gene Expression. Front Immunol. 2021;12: 659761.](http://paperpile.com/b/AlC1NU/ZTlEN)

4.	[Spitz F, Furlong EEM. Transcription factors: from enhancer binding to developmental control. Nat Rev Genet. 2012;13: 613–626.](http://paperpile.com/b/AlC1NU/OuIdN)

5.	[Voss TC, Hager GL. Dynamic regulation of transcriptional states by chromatin and transcription factors. Nat Rev Genet. 2014;15: 69–81.](http://paperpile.com/b/AlC1NU/IQtx5)

6.	[Xia J-H, Wei G-H. Enhancer Dysfunction in 3D Genome and Disease. Cells. 2019;8. doi:](http://paperpile.com/b/AlC1NU/I17sU)[10.3390/cells8101281](http://dx.doi.org/10.3390/cells8101281)

7.	[Stadhouders R, Filion GJ, Graf T. Transcription factors and 3D genome conformation in cell-fate decisions. Nature. 2019;569: 345–354.](http://paperpile.com/b/AlC1NU/SrPK6)

8.	[Rothenberg EV. Logic and lineage impacts on functional transcription factor deployment for T-cell fate commitment. Biophys J. 2021;120: 4162–4181.](http://paperpile.com/b/AlC1NU/sLLn9)

9.	[Hu Z, Tee W-W. Enhancers and chromatin structures: regulatory hubs in gene expression and diseases. Biosci Rep. 2017;37. doi:](http://paperpile.com/b/AlC1NU/wUte9)[10.1042/BSR20160183](http://dx.doi.org/10.1042/BSR20160183)

10.	[Elimelech A, Birnbaum RY. From 3D organization of the genome to gene expression. Curr Opin Syst Biol. 2020;22: 22–31.](http://paperpile.com/b/AlC1NU/ZuF9o)

11.	[Razin SV, Ulianov SV, Iarovaia OV. Enhancer Function in the 3D Genome. Genes (Basel). 2023;14. doi:](http://paperpile.com/b/AlC1NU/LCSaG)[10.3390/genes14061277](http://dx.doi.org/10.3390/genes14061277)

12.	[Karlebach G, Shamir R. Modelling and analysis of gene regulatory networks. Nat Rev Mol Cell Biol. 2008;9: 770–780.](http://paperpile.com/b/AlC1NU/RvpPo)

13.	[Fazilaty H, Rago L, Kass Youssef K, Ocaña OH, Garcia-Asencio F, Arcas A, et al. A gene regulatory network to control EMT programs in development and disease. Nature Communications. 2019;10: 5115.](http://paperpile.com/b/AlC1NU/SrOTI)

14.	[Ray U, Singh A, Samanta D, Dhar R. Gene regulatory network transitions reveal the central transcription factors in lung adenocarcinoma progression. npj Systems Biology and Applications. 2026;12: 18.](http://paperpile.com/b/AlC1NU/vg5HI)

15.	[Itai T, Dai Y, Liu W, Lee D-F, Zhao Z. Investigating prognostic features in high-grade serous ovarian cancer through gene regulatory network inference with single-cell transcriptomic profiles. Scientific Reports. 2025;15: 39345.](http://paperpile.com/b/AlC1NU/G0S8u)

16.	[Nakazawa MA, Tamada Y, Tanaka Y, Ikeguchi M, Higashihara K, Okuno Y. Novel cancer subtyping method based on patient-specific gene regulatory network. Scientific Reports. 2021;11: 23653.](http://paperpile.com/b/AlC1NU/sWAFa)

17.	[Garcia-Alonso L, Holland CH, Ibrahim MM, Turei D, Saez-Rodriguez J. Benchmark and integration of resources for the estimation of human transcription factor activities. Genome Res. 2019;29: 1363–1375.](http://paperpile.com/b/AlC1NU/S1ON8)

18.	[Kolmykov S, Yevshin I, Kulyashov M, Sharipov R, Kondrakhin Y, Makeev VJ, et al. GTRD: an integrated view of transcription regulation. Nucleic Acids Res. 2020;49: D104–D111.](http://paperpile.com/b/AlC1NU/ap2Ao)

19.	[Sandelin A, Alkema W, Engström P, Wasserman WW, Lenhard B. JASPAR: an open‐access database for eukaryotic transcription factor binding profiles. Nucleic Acids Res. 2004;32: D91–D94.](http://paperpile.com/b/AlC1NU/QJOxN)

20.	[Liu Z-P, Wu C, Miao H, Wu H. RegNetwork: an integrated database of transcriptional and post-transcriptional regulatory networks in human and mouse. Database (Oxford). 2015;2015. doi:](http://paperpile.com/b/AlC1NU/r9XLr)[10.1093/database/bav095](http://dx.doi.org/10.1093/database/bav095)

21.	[Hammal F, de Langen P, Bergon A, Lopez F, Ballester B. ReMap 2022: a database of Human, Mouse, Drosophila and Arabidopsis regulatory regions from an integrative analysis of DNA-binding sequencing experiments. Nucleic Acids Res. 2021;50: D316–D325.](http://paperpile.com/b/AlC1NU/PRWZU)

22.	[Han H, Cho JW, Lee S, Yun A, Kim H, Bae D, et al. TRRUST v2: an expanded reference database of human and mouse transcriptional regulatory interactions. Nucleic acids research. 2018;46. doi:](http://paperpile.com/b/AlC1NU/dZZtW)[10.1093/nar/gkx1013](http://dx.doi.org/10.1093/nar/gkx1013)

23.	[Kurup JT, Kim S, Kidder BL. Identifying Cancer Type-Specific Transcriptional Programs through Network Analysis. Cancers (Basel). 2023;15. doi:](http://paperpile.com/b/AlC1NU/bYYdZ)[10.3390/cancers15164167](http://dx.doi.org/10.3390/cancers15164167)

24.	[Yang L, Tu L, Bisht S, Mao Y, Petkovich D, Thursby S-J, et al. Tissue-location-specific transcription programs drive tumor dependencies in colon cancer. Nat Commun. 2024;15: 1384.](http://paperpile.com/b/AlC1NU/OTshK)

25.	[Ben Guebila M, Lopes-Ramos CM, Weighill D, Sonawane AR, Burkholz R, Shamsaei B, et al. GRAND: a database of gene regulatory network models across human conditions. Nucleic Acids Res. 2022;50: D610–D621.](http://paperpile.com/b/AlC1NU/I4I6)

26.	[Giorgi FM. aracne.networks. Bioconductor; 2017. doi:](http://paperpile.com/b/AlC1NU/hepxx)[10.18129/B9.BIOC.ARACNE.NETWORKS](http://dx.doi.org/10.18129/B9.BIOC.ARACNE.NETWORKS)

27.	[Paull EO, Aytes A, Jones SJ, Subramaniam PS, Giorgi FM, Douglass EF, et al. A modular master regulator landscape controls cancer transcriptional identity. Cell. 2021;184: 334–351.e20.](http://paperpile.com/b/AlC1NU/o5XRT)

28.	[Weinstein JN, Collisson EA, Mills GB, Shaw KRM, Ozenberger BA, Ellrott K, et al. The Cancer Genome Atlas Pan-Cancer analysis project. Nature Genetics. 2013;45: 1113–1120.](http://paperpile.com/b/AlC1NU/aPCr)

29.	[Liska O, Bohár B, Hidas A, Korcsmáros T, Papp B, Fazekas D, et al. TFLink: an integrated gateway to access transcription factor–target gene interactions for multiple species. Database (Oxford). 2022;2022: baac083.](http://paperpile.com/b/AlC1NU/zs8ay)

30.	[Colaprico A, Silva TC, Olsen C, Garofano L, Cava C, Garolini D, et al. TCGAbiolinks: an R/Bioconductor package for integrative analysis of TCGA data. Nucleic Acids Res. 2016;44: e71.](http://paperpile.com/b/AlC1NU/fvHny)

31.	[Chen Y, Chen L, Lun ATL, Baldoni PL, Smyth GK. edgeR v4: powerful differential analysis of sequencing data with expanded functionality and improved support for small counts and larger datasets. Nucleic Acids Res. 2025;53: gkaf018.](http://paperpile.com/b/AlC1NU/i7khA)

32.	[Cuesta-Astroz Y, Gischkow Rucatti G, Murgas L, SanMartín CD, Sanhueza M, Martin AJM. Filtering of Data-Driven Gene Regulatory Networks Using as a Case Study. Front Genet. 2021;12: 649764.](http://paperpile.com/b/AlC1NU/BfuBu)

33.	[Hatcher RJ, Dong J, Liu S, Bian G, Contreras A, Wang T, et al. Pttg1/securin is required for the branching morphogenesis of the mammary gland and suppresses mammary tumorigenesis. Proc Natl Acad Sci U S A. 2014;111: 1008–1013.](http://paperpile.com/b/AlC1NU/GiaJY)

34.	[Wei D, Zhan W, Gao Y, Huang L, Gong R, Wang W, et al. RAB31 marks and controls an ESCRT-independent exosome pathway. Cell Res. 2021;31: 157–177.](http://paperpile.com/b/AlC1NU/GfZV3)

35.	[Chen K, Xu J, Tong Y-L, Yan J-F, Pan Y, Wang W-J, et al. Rab31 promotes metastasis and cisplatin resistance in stomach adenocarcinoma through Twist1-mediated EMT. Cell Death Dis. 2023;14: 115.](http://paperpile.com/b/AlC1NU/mz4vD)

36.	[Wu H-Y, Luo L-F, Wei F, Jiang H-M. Comprehensive clinicopathological significance and putative transcriptional mechanisms of Forkhead box M1 factor in hepatocellular carcinoma. World J Surg Oncol. 2023;21: 366.](http://paperpile.com/b/AlC1NU/lfwyX)

37.	[Kim H, Park K-J, Ryu B-K, Park D-H, Kong D-S, Chong K, et al. Forkhead box M1 (FOXM1) transcription factor is a key oncogenic driver of aggressive human meningioma progression. Neuropathol Appl Neurobiol. 2020;46: 125–141.](http://paperpile.com/b/AlC1NU/uV9nF)

38.	[Zheng Y, Guo J, Zhou J, Lu J, Chen Q, Zhang C, et al. FoxM1 transactivates PTTG1 and promotes colorectal cancer cell migration and invasion. BMC Med Genomics. 2015;8: 49.](http://paperpile.com/b/AlC1NU/Ip9YW)

39.	[Zhou C, Wawrowsky K, Bannykh S, Gutman S, Melmed S. E2F1 induces pituitary tumor transforming gene (PTTG1) expression in human pituitary tumors. Mol Endocrinol. 2009;23: 2000–2012.](http://paperpile.com/b/AlC1NU/oZYfH)

40.	[Zhang H, He Z, Qiu L, Wei J, Gong X, Xian M, et al. PRR11 promotes cell proliferation by regulating PTTG1 through interacting with E2F1 transcription factor in pan-cancer. Front Mol Biosci. 2022;9: 877320.](http://paperpile.com/b/AlC1NU/0ml81)

41.	[Lee KJ, Ahn J-H, Kim J-H, Lee YS, Lee J-S, Lee J-H, et al. Non-coding RNA RMRP governs RAB31-dependent MMP secretion, enhancing ovarian cancer invasion. Biochim Biophys Acta Mol Basis Dis. 2025;1871: 167781.](http://paperpile.com/b/AlC1NU/ehTQ)

42.	[Jalagadugula G, Mao G, Goldfinger LE, Wurtzel J, Del Carpio-Cano F, Lambert MP, et al. Defective RAB31-mediated megakaryocytic early endosomal trafficking of VWF, EGFR, and M6PR in RUNX1 deficiency. Blood Adv. 2022;6: 5100–5112.](http://paperpile.com/b/AlC1NU/GEc4N)

43.	[GTEx Consortium. The Genotype-Tissue Expression (GTEx) project. Nat Genet. 2013;45: 580–585.](http://paperpile.com/b/AlC1NU/jAJPI)

44.	[Lachmann A, Torre D, Keenan AB, Jagodnik KM, Lee HJ, Wang L, et al. Massive mining of publicly available RNA-seq data from human and mouse. Nat Commun. 2018;9: 1366.](http://paperpile.com/b/AlC1NU/KbFPk)
